# Supplementary material for: Neutral Frustration Landscape Architecture of SARS-CoV-2 Spike-Antibody Interfaces Shapes Immune Evasion Mechanisms for Ultrapotent Neutralizing Antibodies and Determines Pathways of Viral Adaptation: Insights from Integrative Computational Approach
Source: bioRxiv. 2025 Nov 3:2025.10.31.685956. Preprint. [Version 1] doi: 10.1101/2025.10.31.685956 (PMC12637510; doi:10.1101/2025.10.31.685956)
Supplement: Supplement 1 [file media-1.pdf]

Supplementary Materials

Neutral Frustration Landscape Architecture of SARS-CoV-2 Spike–Antibody Interfaces Shapes Immune Evasion Mechanisms for Ultrapotent Neutralizing Antibodies and Determines Pathways of Viral Adaptation : Insights from Integrative Computational Approach

Mohammed Alshahrani,[1] Vedant Parikh,[1] Brandon Foley,[1] Gennady Verkhivker [1,2,3]*

[1]Keck Center for Science and Engineering, Schmid College of Science and Technology, Chapman University, Orange, CA 92866, United States of America

[2]Department of Biomedical and Pharmaceutical Sciences, Chapman University School of Pharmacy, Irvine, CA 92618, United States of America

[3]Department of Pharmacology, Skaggs School of Pharmacy and Pharmaceutical Sciences, University of California San Diego, 9500 Gilman Drive, La Jolla, CA 92093, United States of America

* Correspondence: verkhivk@chapman.edu; Tel.: +1-714-516-4586 (G.V)

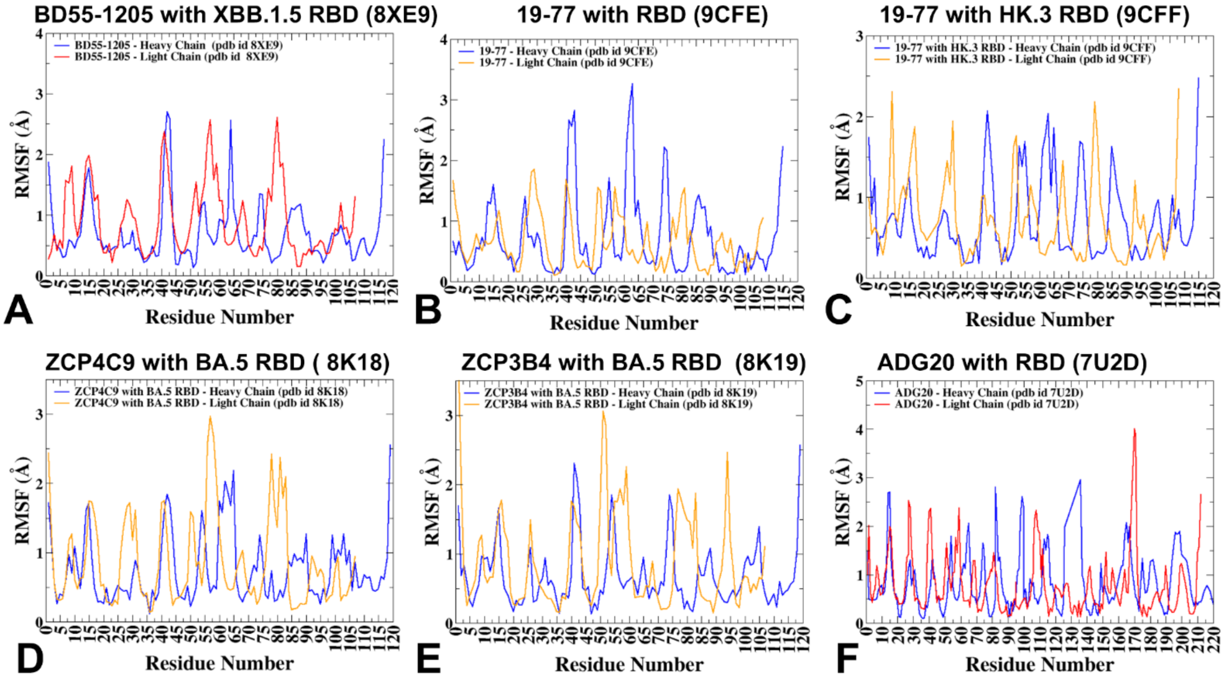

Figure S1. Conformational dynamics profiles obtained from CG-CABS simulations and atomistic reconstruction of the RBD-antibody complexes. The RMSF profiles for the heavy chain residues (in bule lines) and light chain residues (in orange lines) for BD5-1205 obtained from simulations of the S-RBD complexes with BD55-1205, pdb id 8XE9 (A), for 19-77 heavy and light chains with RBD, pdb id 9CFE (B), for 19-77 heavy and light chains with HK.3 RBD, pdb id 9CFF (C), for ZCP4C9 heavy and light chains with BA.5 RBD, pdb id 8K18 (D), for heavy and light chains of ZCP3B4 with BA.5 RBD, pdb id 8K19 (E), and for heavy and light chains of class 4/1 (group F3) ADG20 antibody with RBD , pdb id 7U2D (F).

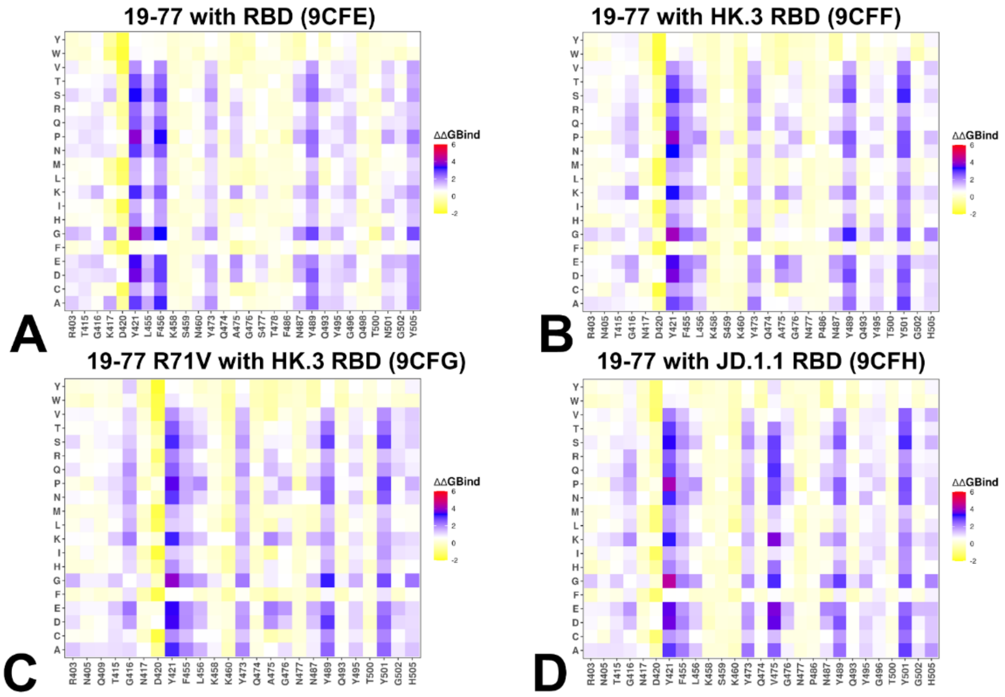

Figure S2. The ensemble-based mutational scanning of binding for the RBD complexes with class 1 19-77 antibody. The mutational scanning heatmaps for the binding epitope residues for 19-77 class 1 antibody with RBD, pdb id 9CFE (A), 19-77 class 1 antibody with HK.3 RBD, pdb id 9CFF (B), 19-77 R71V class 1 antibody with HK.3 RBD, pdb id 9CFG (C), and 19-77 R71V class 1 antibody with JD.1.1 RB, pdb id 9CFH (D). The binding energy hotspots correspond to residues with high mutational sensitivity. The heatmaps show the computed binding free energy changes for 20 single mutations on the sites of variants. The squares on the heatmap are colored using a 4-colored scale blue-white-yellow-red, with blue indicating the largest unfavorable effect

on binding and stability, while yellow-red points to mutations that have favorable effect and improve binding.

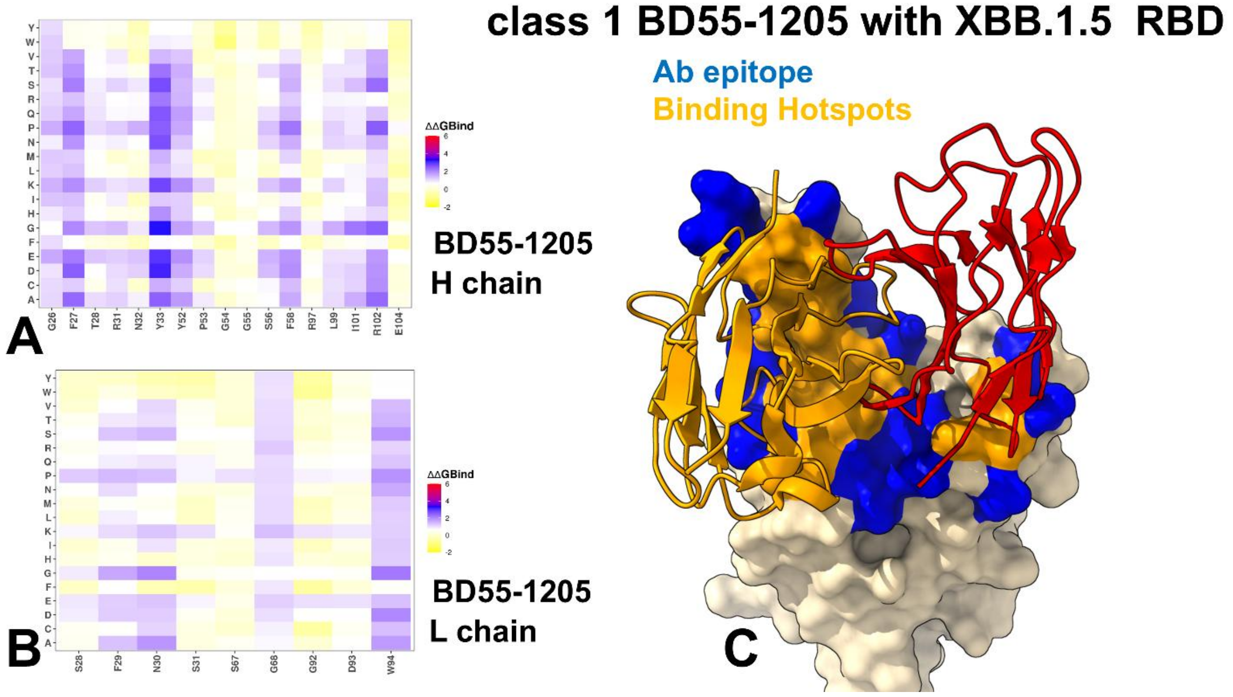

Figure S3. Ensemble-based dynamic mutational profiling of the RBD intermolecular interfaces in the RBD complex with BD55-1205 antibody. The mutational scanning heatmaps are shown for the interfacial heavy chain residues of BD55-1205 (A) and light chain residues of BD55-1205 (B). The heatmaps show the computed binding free energy changes for 20 single mutations of the interfacial positions. (C) The structure of BD55-1205 bound to RBD. The heavy chain of BD55-1205 is in orange ribbons, and light chain is in red ribbons. The binding epitope is shown in blue surface and the positions of the RBD binding energy hotspots are shown in orange-

colored surface. (D). RBD from the complex with BD55-1205. The binding epitope residues are in blue surface and the binding interfacial RBD hotspots are in orange surface.

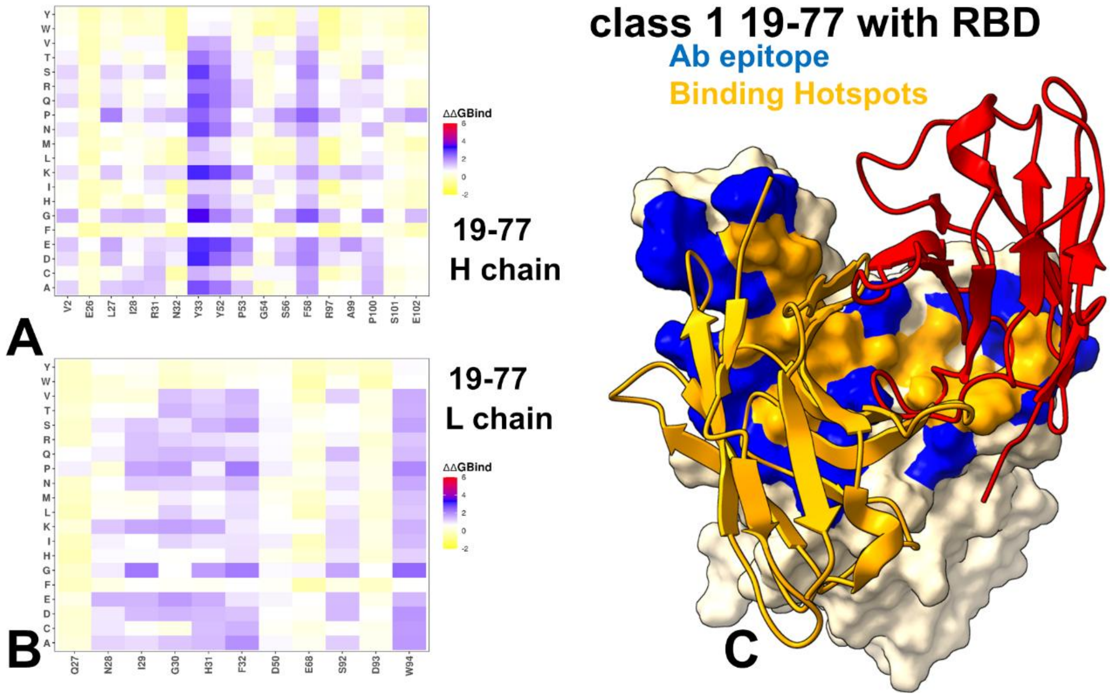

Figure S4. Ensemble-based dynamic mutational profiling of the RBD intermolecular interfaces in the RBD complex with 19-77 antibody. The mutational scanning heatmaps are shown for the interfacial heavy chain residues of 19-77 (A) and light chain residues of 19-77 (B). The heatmaps show the computed binding free energy changes for 20 single mutations of the interfacial positions. (C) The structure of 19-77 bound to RBD. The heavy chain of 19-77 is in orange ribbons, and light chain is in red ribbons. The binding epitope is shown in blue surface and the positions of the RBD binding energy hotspots are shown in orange-colored surface. (D). RBD

from the complex with 19-77. The binding epitope residues are in blue surface and the binding interfacial RBD hotspots are in orange surface.

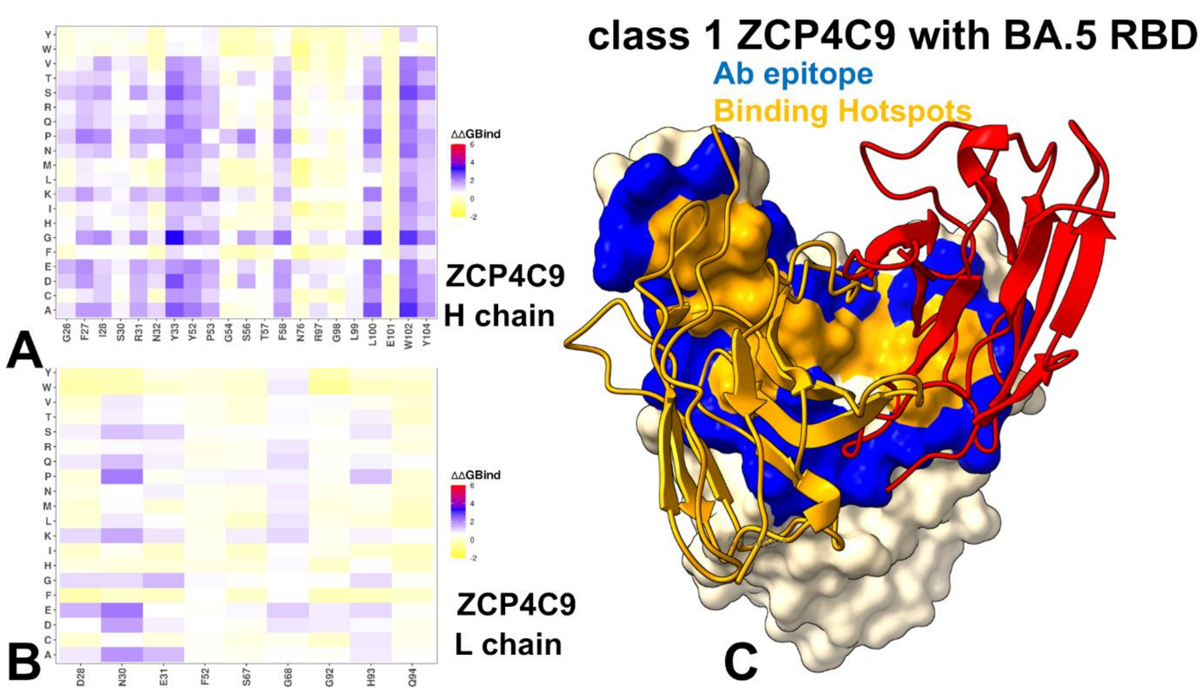

Figure S5. Ensemble-based dynamic mutational profiling of the RBD intermolecular interfaces in the RBD complex with ZCP4C9 antibody. The mutational scanning heatmaps are shown for the interfacial heavy chain residues of ZCP4C9 (A) and light chain residues of

ZCP4C9 (B). The heatmaps show the computed binding free energy changes for 20 single mutations of the interfacial positions. (C) The structure of ZCP4C9 bound to RBD. The heavy chain of ZCP4C9 is in orange ribbons, and light chain is in red ribbons. The binding epitope is shown in blue surface and the positions of the RBD binding energy hotspots are shown in orange-colored surface. (D). RBD from the complex with ZCP4C9. The binding epitope residues are in blue surface and the binding interfacial RBD hotspots are in orange surface.

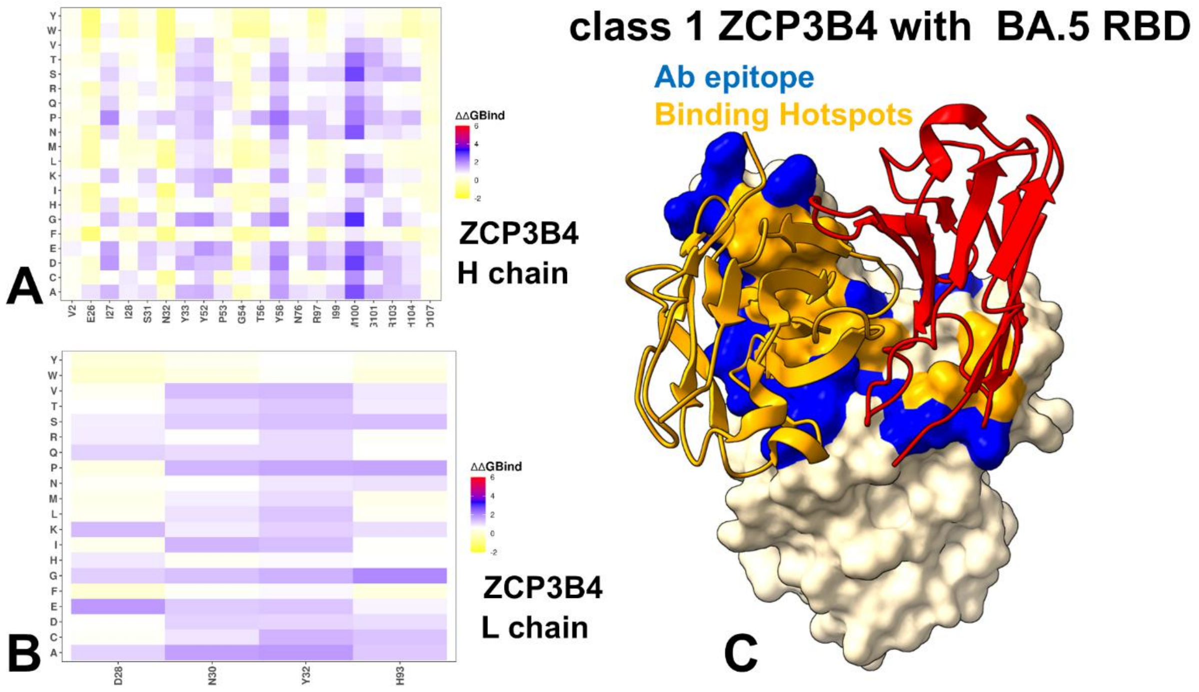

Figure S6. Ensemble-based dynamic mutational profiling of the RBD intermolecular interfaces in the RBD complex with ZCP3B4 antibody. The mutational scanning heatmaps are shown for the interfacial heavy chain residues of ZCP3B4 (A) and light chain residues of

ZCP3B4 (B). The heatmaps show the computed binding free energy changes for 20 single mutations of the interfacial positions. (C) The structure of ZCP3B4 bound to RBD. The heavy chain of ZCP3B4 is in orange ribbons, and light chain is in red ribbons. The binding epitope is shown in blue surface and the positions of the RBD binding energy hotspots are shown in orange-colored surface. (D). RBD from the complex with ZCP3B4. The binding epitope residues are in blue surface and the binding interfacial RBD hotspots are in orange surface.

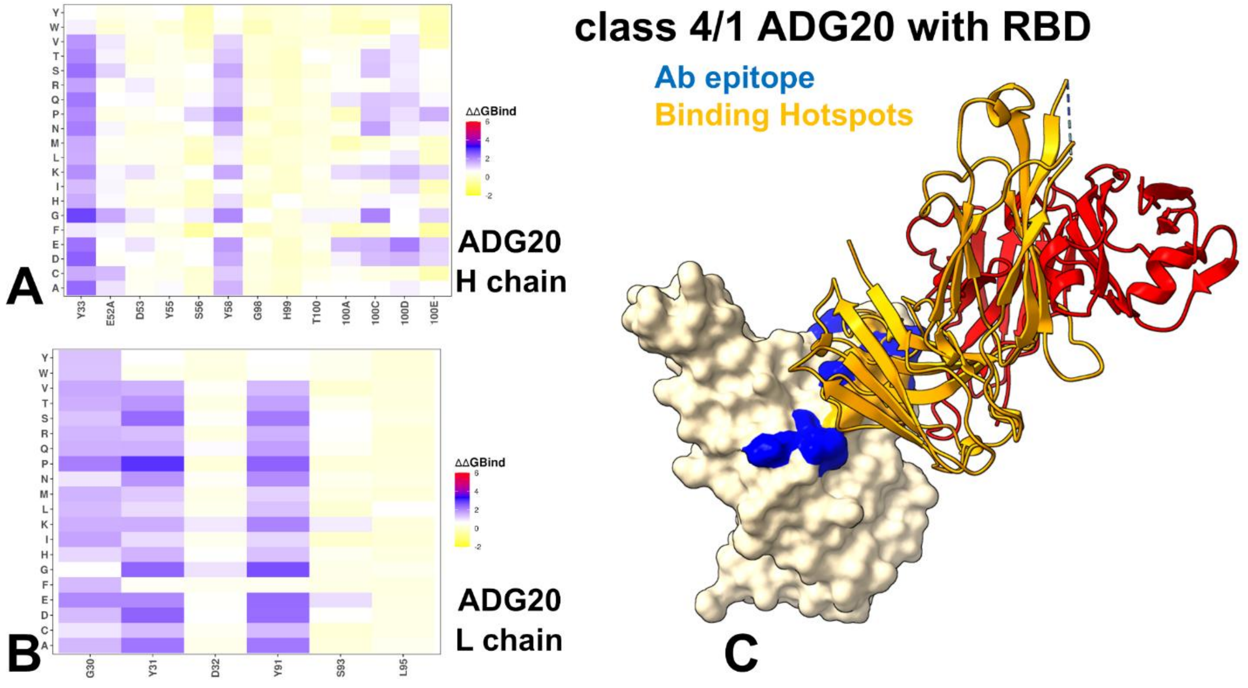

Figure S7. Ensemble-based dynamic mutational profiling of the RBD intermolecular interfaces in the RBD complex with ADG20 antibody. The mutational scanning heatmaps are shown for the interfacial heavy chain residues of ADG20 (A) and light chain residues of ADG20 (B). The heatmaps show the computed binding free energy changes for 20 single mutations of the

interfacial positions. (C) The structure of ADG20 bound to RBD. The heavy chain of ADG20 is in orange ribbons, and light chain is in red ribbons. The binding epitope is shown in blue surface and the positions of the RBD binding energy hotspots are shown in orange-colored surface. (D). RBD from the complex with ADG20. The binding epitope residues are in blue surface and the binding interfacial RBD hotspots are in orange surface.

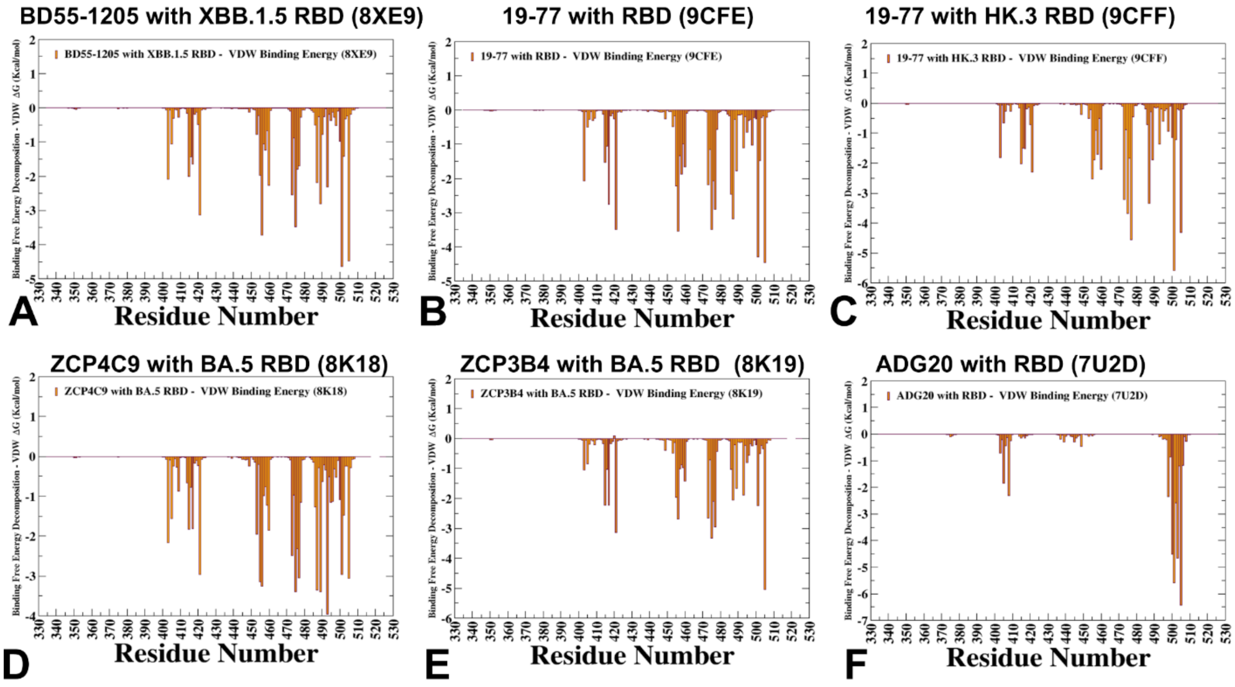

Figure S8. The residue-based decomposition of the van der Waals contribution of MM-GBSA energies for the S-RBD complexes with class 1 BD55-1205 with XBB.1.5 RBD (A), 19-77 class 1 antibody with RBD (B), 19-77 class 1 antibody with HK.3 RBD (C), class 1 ZCP4C9 with

BA.5 RBD (D), class 1 ZCP3B4 with BA.5 RBD (E), class 4/1 antibody ADG20 with RBD (F). The binding free energy with MM-GBSA was computed by averaging the results of computations over 10,000 samples from the equilibrium ensembles.

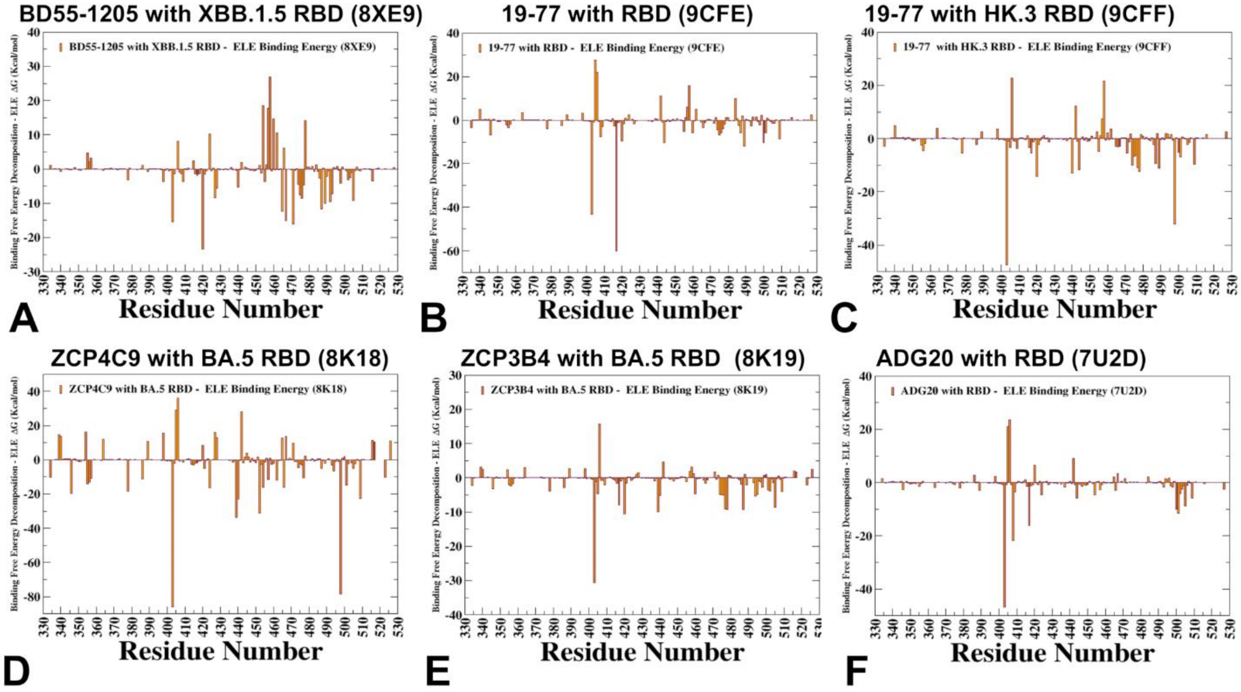

Figure S9. The residue-based decomposition of the electrostatic contribution of MM-GBSA energies for the S-RBD complexes with class 1 BD55-1205 with XBB.1.5 RBD (A), 19-77 class 1 antibody with RBD (B), 19-77 class 1 antibody with HK.3 RBD (C), class 1 ZCP4C9 with BA.5 RBD (D), class 1 ZCP3B4 with BA.5 RBD (E), class 4/1 antibody ADG20 with RBD (F). The binding free energy with MM-GBSA was computed by averaging the results of computations over 10,000 samples from the equilibrium ensembles.

Table S1. Mutational landscape of the Omicron variants.

Variant	Mutational landscape
XBB.1.5	T19I, V83A, G142D, Del144, H146Q, Q183E, V213E, G252V, G339H, R346T, L368I, S371F, S373P, S375F, T376A, D405N, R408S, K417N, N440K, V445P, G446S,N460K, S477N, T478K, E484A, **F486P, F490S**, R493Q reversal, Q498R, N501Y, Y505H, D614G, H655Y, N679K, P681H, N764K, D796Y, Q954H, N969K
JN.1	T19I, R21T, S50L, del69-70,V127F, delY144, F157S, R158G, delN211, L213I, L226F, H25N, A264D, I332V, D339H, K356T, R403K, V445H, G446S, N450D, L452W, **L455S,** N460K, N481K, del V483, A484K, F486P, R493Q, E554K, A570V, P612S, I670V, H68R, D939F, P1143L
KP.2	**JN.1 + S:R346T, S:F456L, S:V1104L**<br><br>T19I, R21T, S50L, del69-70,V127F, delY144, F157S, R158G, delN211, L213I, L226F, H25N,A264D, I332V, D339H, **R346T**, K356T, R403K, V445H, G446S, N450D, L452W, **L455S, F456L**, N460K, N481K, del V483, A484K, F486P, R493Q, E554K, A570V, P612S, I670V, H68R, D939F, **V1104L,** P1143L
KP.3	**JN.1 + S:F456L, S:Q493E, S:V1104L**<br><br>T19I, R21T, S50L, del69-70,V127F, delY144, F157S, R158G, delN211, L213I, L226F, H25N,A264D, I332V, D339H, K356T, R403K, V445H, G446, N450D, L452W, **L455S, F456L**, N460K, N481K, del V483, A484K, F486P, **Q493E**, E554K, A570V, P612S, I670V, H68R, D939F, **V1104L,** P1143L
KP.1.1	**JN.1 + S:F456L, S:R346T, S:K1086R, S:V1104L**<br><br>T19I, R21T, S50L, del69-70,V127F, delY144, F157S, R158G, delN211, L213I, L226F, H25N,A264D, I332V, D339H, **R346T**, K356T, R403K, V445H, G446, N450D, L452W, **L455S, F456L**, N460K, N481K, del V483, A484K, F486P, **Q493E**, E554K, A570V, P612S, H68R, D939F, **K1086R**, **V1104L,** P1143L

KP.3.1.1	**KP.3 + S:S31-**<br><br>T19I, R21T, S31-, S50L, del69-70,V127F, delY144, F157S, R158G, delN211, L213I, L226F, H25N,A264D, I332V, D339H, K356T, R403K, V445H, G446, N450D, L452W, L455S, F456L, N460K, N481K, del V483, A484K, F486P, Q493E, E554K, A570V, P612S, I670V, H68R, D939F, V1104L, P1143L
LP.8	**KP.1.1+ F186L, H445R, Q493E, S31 del**<br><br>T19I, R21T, S31 del, S50L, del69-70,V127F, delY144, F157S, R158G, F186L, delN211, L213I, L226F, H25N,A264D, I332V, D339H, **R346T**, K356T, R403K, H445R, G446, N450D, L452W, **L455S, F456L**, N460K, N481K, del V483, A484K, F486P, **Q493E**, E554K, A570V, P612S, I670V, H68R, D939F, **K1086R**, **V1104L,** P1143L
LB.1	**JN.1+ S:S31-, S:Q183H, S:R346T, S:F456L**<br><br>T19I, R21T, **S31-**, S50L, del69-70,V127F, delY144, F157S, R158G, **Q183H**, delN211, L213I, L226F, H25N,A264D, I332V, D339H, **R346T,** K356T, R403K, V445H, G446S, N450D, L452W, **L455S, F456L**, N460K, N481K, del V483, A484K, F486P, R493Q, E554K, A570V, P612S, I670V, H68R, D939F, P1143L
XEC	**JN.1 + S:T22N, S:F59S, S:F456L, S:Q493E, S:V1104L**<br><br>T19I, R21T, **T22N**, S50L, **F59S**, del69-70,V127F, delY144, F157S, R158G, delN211, L213I, L226F, H25N,A264D, I332V, D339H, K356T, R403K, V445H, G446S, N450D, L452W, **L455S,** F456L. N460K, N481K, del V483, A484K, F486P, **Q493E**, E554K, A570V, P612S, I670V, H68R, D939F, **V1104L**, P1143L
LB.8.1	**JN1 + S:S31-, S:F186L, S:R190S, S:R346T, S:V445R, S:F456L, S:Q493E, S:K1086R, S:V1104L**<br><br>T19I, R21T, S31-, S50L, del69-70,V127F, delY144, F157S, R158G, F186L, R190S, delN211, L213I, L226F, H25N, A264D, I332V, D339H, R346T, K356T, R403K, V445R, G446S, N450D, L452W, **L455S,** F456L, N460K, N481K, del V483, A484K, F486P, Q493E, E554K, A570V, P612S, I670V, H68R, D939F, K1086R, V1140L, P1143L
NB.1.8.1	**JN1 + S:T22N, S:F59S, S:G184S,S:A435S, S:F456L, S:T478I, S:Q493E**
XFG	**JN1 + S:T22N, S:S31P, S:K182R, S:R190S, S:R346T, S:K444R, S:V445R, S:F456L, S:N487D, S:Q493E, S:T572I**

Table S2. The list of the intermolecular contacts in the structure of the Class 1 BD55-1205 antibody complex with XBB.1.5 RBD (pdb id 8XE9). The interfacial contacts in the structure are defined by counting the number of interatomic contacts within a 5.5 Å distance threshold between atoms of the interacting proteins.

**RBD Residue**	**RBD Residue Number**	**RBD chain**	**Ab Residue**	**Ab Residue Number**	**Ab chain**
ARG	403	C	ASN	30	B
ARG	403	C	GLY	92	B
ASN	405	C	ASP	93	B
THR	415	C	SER	56	A
THR	415	C	THR	57	A
THR	415	C	PHE	58	A
GLY	416	C	TYR	52	A
GLY	416	C	SER	56	A
GLY	416	C	PHE	58	A
ASN	417	C	TYR	33	A
ASN	417	C	TYR	52	A
ASN	417	C	TRP	94	B
ASN	417	C	PRO	95	B
ASP	420	C	TYR	52	A
ASP	420	C	SER	56	A
TYR	421	C	TYR	33	A
TYR	421	C	TYR	52	A
TYR	421	C	PRO	53	A
TYR	421	C	GLY	54	A
TYR	421	C	GLY	55	A
TYR	453	C	ILE	101	A
LEU	455	C	TYR	33	A
LEU	455	C	PRO	53	A
LEU	455	C	TRP	94	B

LEU	455	C	LEU	99	A
LEU	455	C	ILE	101	A
LEU	455	C	ARG	102	A
PHE	456	C	ARG	31	A
PHE	456	C	ASN	32	A
PHE	456	C	TYR	33	A
PHE	456	C	PRO	53	A
PHE	456	C	LEU	99	A
ARG	457	C	PRO	53	A
ARG	457	C	GLY	54	A
LYS	458	C	SER	30	A
LYS	458	C	ARG	31	A
LYS	458	C	PRO	53	A
LYS	458	C	GLY	54	A
SER	459	C	PRO	53	A
SER	459	C	GLY	54	A
LYS	460	C	GLY	54	A
LYS	460	C	GLY	55	A
LYS	460	C	SER	56	A
TYR	473	C	SER	30	A
TYR	473	C	ARG	31	A
TYR	473	C	ASN	32	A
TYR	473	C	PRO	53	A
GLN	474	C	ARG	31	A
ALA	475	C	PHE	27	A
ALA	475	C	THR	28	A
ALA	475	C	ARG	31	A
ALA	475	C	ASN	32	A
ALA	475	C	ARG	97	A
GLY	476	C	GLY	26	A
GLY	476	C	PHE	27	A
GLY	476	C	THR	28	A
GLY	476	C	ARG	31	A
GLY	476	C	ASN	32	A
ASN	477	C	GLY	26	A
ASN	477	C	PHE	27	A
ASN	477	C	THR	28	A
PRO	486	C	GLU	104	A
ASN	487	C	VAL	2	A
ASN	487	C	GLY	26	A
ASN	487	C	PHE	27	A
ASN	487	C	ARG	97	A
ASN	487	C	GLU	104	A

TYR	489	C	ASN	32	A
TYR	489	C	ARG	97	A
TYR	489	C	LEU	99	A
TYR	489	C	ARG	102	A
TYR	489	C	GLU	104	A
SER	490	C	ARG	102	A
PRO	491	C	ARG	102	A
LEU	492	C	ARG	102	A
GLN	493	C	ILE	101	A
GLN	493	C	ARG	102	A
ARG	498	C	SER	31	B
ARG	498	C	SER	67	B
THR	500	C	SER	28	B
THR	500	C	PHE	29	B
THR	500	C	GLY	68	B
TYR	501	C	SER	28	B
TYR	501	C	PHE	29	B
TYR	501	C	ASN	30	B
TYR	501	C	SER	31	B
GLY	502	C	SER	28	B
GLY	502	C	PHE	29	B
GLY	502	C	ASN	30	B
VAL	503	C	SER	28	B
HIS	505	C	SER	28	B
HIS	505	C	PHE	29	B
HIS	505	C	ASN	30	B
HIS	505	C	GLY	92	B
HIS	505	C	ASP	93	B

Table S3. The list of the intermolecular contacts in the structure of the Class 1 19-77 antibody complex with RBD (pdb id 9CFE). The interfacial contacts in the structure are defined by counting the number of interatomic contacts within a 5.5 Å distance threshold between atoms of the interacting proteins.

RBD Residue	RBD Residue Number	RBD chain	Ab Residue	Ab Residue Number	Ab chain
ARG	403	C	PHE	32	B
ARG	403	C	SER	92	B
ARG	403	C	ASP	93	B
ASP	405	C	SER	92	B
ARG	408	C	PHE	58	A
THR	415	C	SER	56	A
THR	415	C	PHE	58	A
GLY	416	C	TYR	52	A
GLY	416	C	SER	56	A
GLY	416	C	PHE	58	A
LYS	417	C	TYR	33	A
LYS	417	C	TYR	52	A
LYS	417	C	ASP	93	B
LYS	417	C	TRP	94	B
LYS	417	C	PRO	95	B
ASP	420	C	TYR	52	A
ASP	420	C	SER	56	A
ASP	420	C	PHE	58	A
TYR	421	C	TYR	33	A
TYR	421	C	TYR	52	A
TYR	421	C	PRO	53	A
TYR	421	C	GLY	54	A
TYR	421	C	GLY	55	A
TYR	421	C	SER	56	A

LEU	455	C	TYR	33	A
LEU	455	C	PRO	53	A
LEU	455	C	TRP	94	B
LEU	455	C	PRO	100	A
PHE	456	C	ARG	31	A
PHE	456	C	ASN	32	A
PHE	456	C	TYR	33	A
PHE	456	C	PRO	53	A
PHE	456	C	TRP	94	B
PHE	456	C	ASP	98	A
PHE	456	C	ALA	99	A
PHE	456	C	PRO	100	A
ARG	457	C	ARG	31	A
ARG	457	C	PRO	53	A
ARG	457	C	GLY	54	A
LYS	458	C	SER	30	A
LYS	458	C	ARG	31	A
LYS	458	C	PRO	53	A
LYS	458	C	GLY	54	A
LYS	458	C	ARG	71	A
SER	459	C	PRO	53	A
SER	459	C	GLY	54	A
ASN	460	C	PRO	53	A
ASN	460	C	GLY	54	A
ASN	460	C	GLY	55	A
ASN	460	C	SER	56	A
TYR	473	C	SER	30	A
TYR	473	C	ARG	31	A
TYR	473	C	ASN	32	A
TYR	473	C	PRO	53	A
GLN	474	C	ILE	28	A
GLN	474	C	ARG	31	A
ALA	475	C	GLU	26	A
ALA	475	C	LEU	27	A
ALA	475	C	ILE	28	A
ALA	475	C	ARG	31	A
ALA	475	C	ASN	32	A
ALA	475	C	ARG	97	A
GLY	476	C	GLU	26	A
GLY	476	C	LEU	27	A
GLY	476	C	ILE	28	A
GLY	476	C	ASN	32	A
SER	477	C	SER	25	A

SER	477	C	GLU	26	A
SER	477	C	LEU	27	A
SER	477	C	ILE	28	A
THR	478	C	GLU	26	A
GLY	485	C	GLU	102	A
PHE	486	C	GLU	1	A
PHE	486	C	VAL	2	A
PHE	486	C	GLU	26	A
PHE	486	C	GLU	102	A
ASN	487	C	VAL	2	A
ASN	487	C	GLU	26	A
ASN	487	C	LEU	27	A
ASN	487	C	ARG	97	A
ASN	487	C	GLU	102	A
ASN	487	C	ASP	104	A
TYR	489	C	ASN	32	A
TYR	489	C	ARG	97	A
TYR	489	C	ALA	99	A
TYR	489	C	PRO	100	A
TYR	489	C	SER	101	A
TYR	489	C	GLU	102	A
GLN	493	C	ASP	50	B
GLN	493	C	PRO	100	A
TYR	495	C	HIS	31	B
TYR	495	C	PHE	32	B
GLY	496	C	HIS	31	B
GLN	498	C	HIS	31	B
PRO	499	C	GLU	68	B
THR	500	C	ASN	28	B
THR	500	C	GLU	68	B
ASN	501	C	ASN	28	B
ASN	501	C	ILE	29	B
ASN	501	C	GLY	30	B
ASN	501	C	HIS	31	B
ASN	501	C	SER	67	B
ASN	501	C	GLU	68	B
GLY	502	C	ASN	28	B
GLY	502	C	GLY	30	B
GLY	502	C	GLU	68	B
TYR	505	C	ILE	2	B
TYR	505	C	GLN	27	B
TYR	505	C	ASN	28	B
TYR	505	C	ILE	29	B

TYR	505	C	GLY	30	B
TYR	505	C	PHE	32	B
TYR	505	C	GLU	90	B
TYR	505	C	SER	92	B

Table S4. The list of the intermolecular contacts in the structure of the Class 1 ZCP4C9 antibody complex with BA.5 RBD (pdb id 8K18). The interfacial contacts in the structure are defined by counting the number of interatomic contacts within a 5.5 Å distance threshold between atoms of the interacting proteins.

RBD Residue	RBD Residue Number	RBD chain	Ab Residue	Ab Residue Number	Ab chain
LYS	403	E	ASN	30	D
LYS	403	E	TRP	102	C
ASP	405	E	GLY	92	D
ASP	405	E	HIS	93	D
SER	408	E	GLN	94	D
GLN	409	E	GLN	94	D
GLN	414	E	GLN	94	D
THR	415	E	SER	56	C
THR	415	E	THR	57	C
THR	415	E	PHE	58	C
GLY	416	E	SER	56	C
GLY	416	E	PHE	58	C
GLY	416	E	GLN	94	D
ASN	417	E	TYR	33	C
ASN	417	E	TYR	52	C
ASN	417	E	PHE	58	C
ASP	420	E	GLY	55	C
ASP	420	E	SER	56	C
TYR	421	E	TYR	33	C
TYR	421	E	TYR	52	C
TYR	421	E	PRO	53	C
TYR	421	E	GLY	54	C
TYR	421	E	GLY	55	C
TYR	421	E	SER	56	C
TYR	453	E	TRP	102	C

TYR	453	E	TYR	104	C
LEU	455	E	TYR	33	C
LEU	455	E	PRO	53	C
LEU	455	E	LEU	99	C
LEU	455	E	GLU	101	C
LEU	455	E	TYR	104	C
PHE	456	E	ARG	31	C
PHE	456	E	TYR	33	C
PHE	456	E	PRO	53	C
PHE	456	E	LEU	99	C
PHE	456	E	LEU	100	C
ARG	457	E	PRO	53	C
ARG	457	E	GLY	54	C
LYS	458	E	ARG	31	C
LYS	458	E	PRO	53	C
LYS	458	E	GLY	54	C
SER	459	E	PRO	53	C
SER	459	E	GLY	54	C
ASN	460	E	GLY	54	C
ASN	460	E	GLY	55	C
ASN	460	E	SER	56	C
TYR	473	E	SER	30	C
TYR	473	E	ARG	31	C
TYR	473	E	ASN	32	C
TYR	473	E	PRO	53	C
TYR	473	E	GLY	54	C
GLN	474	E	ARG	31	C
ALA	475	E	ILE	28	C
ALA	475	E	VAL	29	C
ALA	475	E	SER	30	C
ALA	475	E	ARG	31	C
ALA	475	E	ASN	32	C
GLY	476	E	PHE	27	C
GLY	476	E	ILE	28	C
GLY	476	E	SER	30	C
GLY	476	E	ARG	31	C
GLY	476	E	ASN	32	C
ASN	477	E	ILE	28	C
ASN	477	E	SER	30	C
ASN	477	E	ARG	31	C
ASN	477	E	ASN	76	C
LYS	478	E	ILE	28	C
VAL	486	E	GLY	26	C

ASN	487	E	GLY	26	C
ASN	487	E	PHE	27	C
ASN	487	E	ILE	28	C
ASN	487	E	ASN	32	C
TYR	489	E	PHE	27	C
TYR	489	E	ASN	32	C
TYR	489	E	ARG	97	C
TYR	489	E	GLY	98	C
TYR	489	E	LEU	99	C
TYR	489	E	LEU	100	C
PHE	490	E	LEU	100	C
GLN	493	E	LEU	99	C
GLN	493	E	LEU	100	C
GLN	493	E	GLU	101	C
GLN	493	E	TRP	102	C
GLN	493	E	TYR	104	C
SER	494	E	TRP	102	C
TYR	495	E	TRP	102	C
SER	496	E	GLU	31	D
SER	496	E	TRP	102	C
ARG	498	E	GLU	31	D
ARG	498	E	PHE	52	D
THR	500	E	ASP	28	D
THR	500	E	GLU	31	D
THR	500	E	SER	67	D
THR	500	E	GLY	68	D
TYR	501	E	ASP	28	D
TYR	501	E	ASN	30	D
TYR	501	E	GLU	31	D
TYR	501	E	GLY	68	D
GLY	502	E	ASP	28	D
GLY	502	E	ASN	30	D
GLY	502	E	GLY	68	D
VAL	503	E	ASP	28	D
GLY	504	E	ASP	28	D
HIS	505	E	ASP	28	D
HIS	505	E	VAL	29	D
HIS	505	E	ASN	30	D
HIS	505	E	ASP	32	D
HIS	505	E	GLY	92	D
HIS	505	E	HIS	93	D
GLN	506	E	ASN	30	D

Table S5. The list of the intermolecular contacts in the structure of the Class 1 ZCP3B4 antibody complex with BA.5 RBD (pdb id 8K19). The interfacial contacts in the structure are defined by counting the number of interatomic contacts within a 5.5 Å distance threshold between atoms of the interacting proteins.

RBD Residue	RBD Residue Number	RBD chain	Ab Residue	Ab Residue Number	Ab chain
LYS	403	E	ASP	92	B
LYS	403	E	HIS	93	B
ASN	405	E	HIS	93	B
ASP	406	E	HIS	93	B
THR	415	E	TYR	52	A
THR	415	E	THR	56	A
THR	415	E	THR	57	A
THR	415	E	TYR	58	A
GLY	416	E	TYR	52	A
GLY	416	E	THR	56	A
GLY	416	E	TYR	58	A
ASN	417	E	TYR	33	A
ASN	417	E	TYR	52	A
ASN	417	E	TYR	58	A
ASP	420	E	TYR	52	A
ASP	420	E	THR	56	A
ASP	420	E	TYR	58	A
TYR	421	E	TYR	33	A
TYR	421	E	TYR	52	A
TYR	421	E	PRO	53	A
TYR	421	E	GLY	54	A
TYR	421	E	GLY	55	A
TYR	421	E	MET	100	A
TYR	449	E	ARG	103	A
TYR	453	E	TYR	32	B
TYR	453	E	GLY	101	A

TYR	453	E	ARG	103	A
LEU	455	E	MET	100	A
LEU	455	E	GLY	101	A
LEU	455	E	GLY	102	A
PHE	456	E	MET	100	A
PHE	456	E	GLY	101	A
ARG	457	E	PRO	53	A
ARG	457	E	GLY	54	A
ARG	457	E	MET	100	A
LYS	458	E	SER	30	A
LYS	458	E	SER	31	A
LYS	458	E	PRO	53	A
LYS	458	E	GLY	54	A
SER	459	E	SER	30	A
SER	459	E	PRO	53	A
SER	459	E	GLY	54	A
ASN	460	E	PRO	53	A
ASN	460	E	GLY	54	A
ASN	460	E	GLY	55	A
ASN	460	E	THR	56	A
TYR	473	E	SER	30	A
TYR	473	E	SER	31	A
TYR	473	E	ASN	32	A
TYR	473	E	PRO	53	A
TYR	473	E	MET	100	A
GLN	474	E	ILE	28	A
GLN	474	E	SER	31	A
ALA	475	E	GLU	26	A
ALA	475	E	ILE	27	A
ALA	475	E	ILE	28	A
ALA	475	E	VAL	29	A
ALA	475	E	SER	31	A
ALA	475	E	ASN	32	A
ALA	475	E	ARG	97	A
GLY	476	E	GLU	26	A
GLY	476	E	ILE	27	A
GLY	476	E	ILE	28	A
GLY	476	E	SER	31	A
GLY	476	E	ASN	32	A
ASN	477	E	GLU	26	A
ASN	477	E	ILE	27	A
ASN	477	E	ILE	28	A
ASN	477	E	ASN	76	A

LYS	478	E	GLU	26	A
VAL	486	E	VAL	2	A
VAL	486	E	GLU	26	A
ASN	487	E	GLU	26	A
ASN	487	E	ILE	27	A
TYR	489	E	ARG	97	A
TYR	489	E	ILE	99	A
TYR	489	E	HIS	104	A
TYR	489	E	ASP	107	A
GLN	493	E	MET	100	A
GLN	493	E	GLY	101	A
GLN	493	E	GLY	102	A
GLN	493	E	ARG	103	A
GLN	493	E	HIS	104	A
SER	494	E	ARG	103	A
TYR	495	E	ARG	103	A
SER	496	E	ARG	103	A
TYR	501	E	ASP	28	B
TYR	501	E	ASN	30	B
GLY	502	E	ASP	28	B
VAL	503	E	ASP	28	B
GLY	504	E	ASP	28	B
GLY	504	E	HIS	93	B
HIS	505	E	ASP	28	B
HIS	505	E	ILE	29	B
HIS	505	E	ASN	30	B
HIS	505	E	TYR	32	B
HIS	505	E	ASP	92	B
HIS	505	E	HIS	93	B

Table S6. The list of the intermolecular contacts in the structure of the Class 4/1 ADG20 antibody complex with BA.5 RBD (pdb id 7U2D). The interfacial contacts in the structure are defined by counting the number of interatomic contacts within a 5.5 Å distance threshold between atoms of the interacting proteins.

RBD Residue	RBD Residue Number	RBD chain	Ab Residue	Ab Residue Number	Ab chain
ARG	403	A	TYR	33	H
ASP	405	A	TYR	33	H
ASP	405	A	SER	52	H
ASP	405	A	ASP	53	H
ASP	405	A	SER	56	H
ASP	405	A	TYR	58	H
ARG	408	A	ASP	53	H
ARG	408	A	TYR	55	H
ARG	408	A	SER	56	H
GLN	409	A	ASP	53	H
ASN	439	A	TYR	31	L
TYR	449	A	HIS	99	H
GLY	496	A	GLY	98	H
GLN	498	A	GLY	98	H
GLN	498	A	HIS	99	H
GLN	498	A	THR	100	H
PRO	499	A	GLY	30	L
PRO	499	A	TYR	31	L
THR	500	A	GLY	30	L
THR	500	A	TYR	31	L
THR	500	A	ASP	32	L
THR	500	A	TRP	100	H
ASN	501	A	TYR	31	L
ASN	501	A	TYR	91	L

ASN	501	A	GLY	98	H
ASN	501	A	THR	100	H
ASN	501	A	TRP	100	H
ASN	501	A	GLY	100	H
GLY	502	A	TYR	31	L
GLY	502	A	TYR	91	L
GLY	502	A	TRP	100	H
GLY	502	A	GLY	100	H
VAL	503	A	TYR	31	L
VAL	503	A	TYR	58	H
VAL	503	A	TYR	91	L
VAL	503	A	SER	93	L
VAL	503	A	LEU	95	L
VAL	503	A	GLY	100	H
GLY	504	A	TYR	33	H
GLY	504	A	TYR	58	H
GLY	504	A	TYR	91	L
TYR	505	A	SER	31	H
TYR	505	A	TYR	32	H
TYR	505	A	TYR	33	H
TYR	505	A	PHE	96	H
TYR	505	A	SER	97	H
TYR	505	A	GLY	98	H
TYR	505	A	GLY	100	H
GLN	506	A	TYR	31	L
GLN	506	A	TYR	91	L
GLN	506	A	SER	93	L
